# A novel method for expansion and differentiation of mouse tracheal epithelial cells in culture

**DOI:** 10.1038/s41598-018-25799-6

**Published:** 2018-05-09

**Authors:** Evelien Eenjes, Tinne C. J. Mertens, Marjon J. Buscop-van Kempen, Yolanda van Wijck, Christian Taube, Robbert J. Rottier, Pieter S. Hiemstra

**Affiliations:** 1 Department of Pediatric Surgery, Erasmus Medical Center-Sophia Children’s Hospital, Rotterdam, The Netherlands; 2 Department of Pulmonology, Leiden University Medical Center, Leiden, The Netherlands; 3 Present Address: Department of Biochemistry and Molecular Biology, The University of Texas Health Science Center at Houston, Houston, Texas USA; 4 Present Address: Department of Pulmonary Medicine, West German Lung Center, Essen University Hospital, Ruhrlandklinik, University Duisburg-Essen, Essen, Germany

## Abstract

Air-liquid interface (ALI) cultures of mouse tracheal epithelial cells (MTEC) are a well-established model to study airway epithelial cells, but current methods require large numbers of animals which is unwanted in view of the 3R principle and introduces variation. Moreover, stringent breeding schemes are frequently needed to generate sufficient numbers of genetically modified animals. Current protocols do not incorporate expansion of MTEC, and therefore we developed a protocol to expand MTEC while maintaining their differentiation capacity. MTEC were isolated and expanded using the ROCK inhibitor Y-27632 in presence or absence of the γ-secretase inhibitor DAPT, a Notch pathway inhibitor. Whereas MTEC proliferated without DAPT, growth rate and cell morphology improved in presence of DAPT. ALI-induced differentiation of expanded MTEC resulted in an altered capacity of basal cells to differentiate into ciliated cells, whereas IL-13-induced goblet cell differentiation remained unaffected. Ciliated cell differentiation improved by prolonging the ALI differentiation or by adding DAPT, suggesting that basal cells retain their ability to differentiate. This technique using expansion of MTEC and subsequent ALI differentiation drastically reduces animal numbers and costs for *in vitro* experiments, and will reduce biological variation. Additionally, we provide novel insights in the dynamics of basal cell populations *in vitro*.

## Introduction

Airway epithelial cells play a pivotal role in protecting the lung by acting both as a mechanical and an immunological barrier. The epithelial cells of the upper respiratory tract form a pseudostratified epithelial layer consisting of several epithelial cell types that constitute an efficient host defense system that employs a variety of mechanisms, including its ability to clear inhaled particles and pathogens from the lung using mucociliary clearance. Efficient mucociliary clearance depends on a proper balance between basal, ciliated and secretory cells. The relative distribution of these cell types in the epithelial layer varies depending on the anatomic location within the conducting airways. Altered composition of these epithelial cell types has been implied in several chronic lung diseases, including asthma and chronic obstructive pulmonary disorder (COPD)^1–3^.

Air-liquid interface (ALI) cultures of human primary airway epithelial cells (AEC) are a well-established *in vitro* model to investigate the role of airway epithelial cells in chronic lung diseases^4,5^. Primary AEC are isolated from bronchial biopsies, brushes or resected lung tissue, and can either be cultured directly onto transwell inserts or the cells can first be expanded *in vitro* for subsequent experimental use. AEC freshly isolated from lung tissue consist of multiple cell types, but during *in vitro* culture under submerged conditions the main population that will expand is the basal cells, the epithelial progenitor population^6,7^. Following *in vitro* expansion, primary AEC can be cultured on transwell inserts to establish ALI cultures. To this end, once the cultures have reached full confluence the apical medium is removed to induce an ALI that allows AEC to differentiate into a pseudostratified epithelial layer containing basal, ciliated and secretory cells^8^. Culturing primary airway epithelial cells at ALI provides a platform to investigate not only fully differentiated epithelial layers, but also the mechanisms of differentiation following airway epithelium damage and the dynamic processes of repair after injury^5^. Importantly, ALI cultures allow us to study the effect of airborne exposures on airway epithelial cells, e.g. whole cigarette smoke exposure^9^.

In addition to primary human AEC, various research groups are using cultures of mouse tracheal epithelial cells (MTEC)^7^. These offer the opportunity to closely link *in vitro* and *in vivo* experiments, and make use of the large variety of transgenic mouse lines available. However, it is difficult to maintain MTEC in a proliferative state after isolation, and therefore MTEC are cultured directly onto transwell inserts without prior *in vitro* expansion. As a result, large animal numbers are needed to obtain adequate cell numbers for *in vitro* experiments. Therefore, novel methods are required to subculture MTEC in order to achieve a drastic reduction in animal numbers needed for experiments.

Expanding the progenitor cell population is essential to subculture MTEC. Basal epithelial cells are considered as the progenitor cell type for the maintenance of a pseudostratified airway epithelium of the upper respiratory tract^6^. The mechanisms that control progenitor cell renewal and differentiation to maintain the airway epithelium are still being uncovered, mostly owing to the complex cell-cell interactions and subsequent signaling involved in the decision making towards a specific cell fate. Notch signaling has been implied in the regulation of basal cell self-renewal and differentiation towards the specialized cell types of the epithelial layer. Importantly, inhibition of Notch signaling has been shown to allow expansion of the basal cell population^10–12^.

To investigate the possibility of expanding MTEC while retaining the ability to differentiate, we have developed an alternative culture method that will lead to a drastic reduction in animal numbers needed for *in vitro* experiments. Moreover, subculturing MTEC would allow for increased numbers of *in vitro* experiments without using additional difficult-to-breed transgenic mice. To this end, we have used a combination of Notch signaling inhibition together with adaptation of existing cell culture methods to explore the possibility of subculturing MTEC and subsequent ALI differentiation. Additionally, we also investigated the effect of passaging MTEC on the basal cell type population as these cells are essential for subsequent differentiation into a pseudostratified epithelial layer consisting of multiple epithelial cell types.

## Results

### Serum free medium and the inhibition of Notch signaling enables basal cell expansion

**Figure 1:**
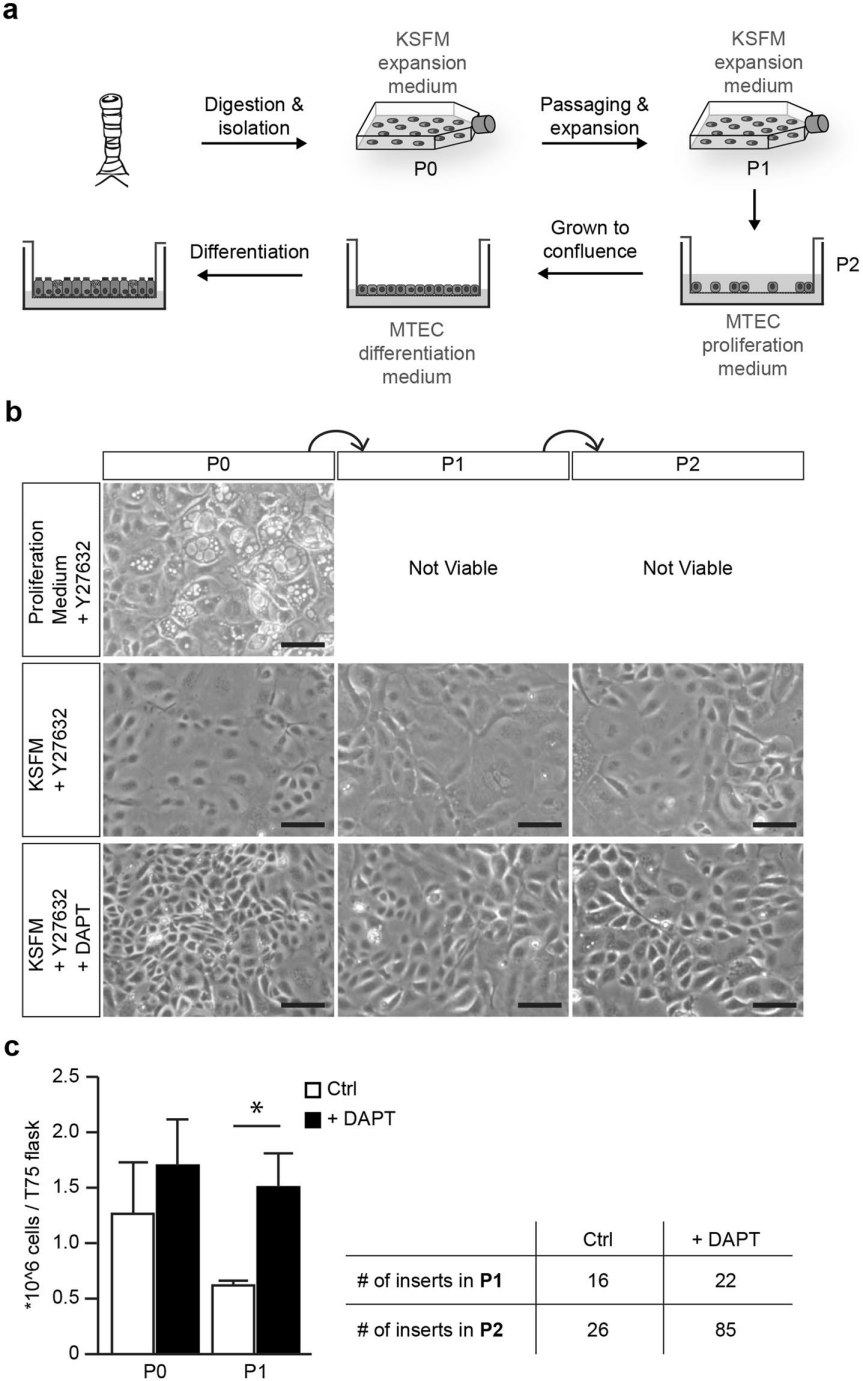
Expanding MTEC in KSFM expansion medium with Notch signaling inhibitor. (**a**) MTEC are expanded to passage 1 in KSFM expansion medium followed by passaging and culturing on transwell inserts. MTEC are grown to full confluence in MTEC proliferation medium, followed by air-liquid interface differentiation in MTEC differentiation medium. (**b**) Serial expansion of MTEC in different medium conditions. Epithelial cells are cultured in proliferation medium with ROCK inhibitor (Y27632), in KSFM medium with ROCK inhibitor or KSFM medium with ROCK inhibitor (Y27632) and γ-secretase inhibitor (DAPT). Phase-contrast images of the MTEC at various passages showing the morphology of the cells. Scale bar, 100 µm. (**c**) The graph represents the number of cells obtained after passaging when cultured in KSFM with ROCK inhibitor alone or in the presence of DAPT (mean ± SEM). *p < 0.05 by unpaired t-test (n = 5). The table shows the number of 12-well inserts that can be obtained after each passage.

Although the current methods of ALI culture represent the pseudostratified airway epithelium *in vitro*, a limitation of this technique is the relatively low number of cells obtained from a trachea. We first validated the existing culturing method of MTEC whereby isolated MTEC are cultured directly onto transwell inserts (Fig. S1). This culture method allowed us to plate approximately 4 to 5 transwell inserts (50,000 cells per insert) using two mice (±100,000 cells per trachea). Improving the expansion of MTEC will allow for multiple experiments to be performed with fewer mice. Ideally, following isolation from the trachea, cells can be expanded in culture for at least two passages to generate sufficient cells for multiple experiments (Fig. 1a). In previous studies, MTEC are isolated and plated on inserts in proliferation medium and upon reaching confluency, cultured at ALI in differentiation medium (Table S1)^13,14^. Following expansion in proliferation medium, MTEC showed a limited proliferation capacity, with the presence of swollen cells with enlarged vacuoles (Fig. 1b). We were unable to successfully passage MTEC grown in proliferation medium. Because we considered the possibility that proliferation medium may be deficient in factors preventing senescence, we switched to another medium. Keratinocyte Serum Free medium supplemented with epithelial growth factor (EGF), Bovine pituitary extract (BPE) and isoproterenol (KSFM) allows for expansion of human airway epithelial cells *in vitro*^15,16^. Additionally, adding a selective inhibitor of Rho-associated, coiled-coil containing protein kinase (ROCK), Y-27632, to the culture medium has been shown to increase proliferation of airway epithelial cells^17^. Growing MTEC in KSFM with Y-27632 indeed resulted in improved cellular expansion, survival and morphology (Fig. 1b). However, despite improved cell morphology and survival, the expansion rate was very slow and still a large number of enlarged cells were present. Notch signaling has been shown to play a pivotal role in preserving the basal cell population and in subsequent differentiation into specialized cell types. Furthermore, inhibition of Notch signaling has been shown to lead to an increased number of basal cells in mouse ALI cultures^10–12,18,19^. To evaluate whether inhibition of Notch signaling promotes the expansion of MTEC after isolation, we added DAPT, a γ-secretase inhibitor and indirect Notch signaling inhibitor, to the KSFM medium containing Y-27632. Growing MTEC in the presence of DAPT and Y-27632 in KSFM medium resulted in a marked increase in proliferation rate with a slightly better morphology compared to cells grown in absence of DAPT; these differences were already apparent after 5 days of culture of P0 cells, but even more after passaging whereby an increased number of transwell inserts could be obtained when DAPT was present during expansion (Fig. 1c). KSFM medium containing Y-27632 and DAPT will be further referred to as KSFM expansion medium.

Notably, expanding MTEC from two mice in KSFM expansion medium resulted in 42.5 million cells, sufficient for 85 transwell inserts when plated at a density of 8 × 10^4^ cells/cm^2^, which is in stark contrast to the 200,000 cells required for 4–5 transwell inserts that are obtained if MTEC were isolated and cultured directly onto the transwell inserts. Also, no contaminating fibroblasts were observed during MTEC expansion. Taken together, we conclude that MTEC expansion is more efficient in the presence of the Notch signaling inhibitor DAPT than without.

### Subcultured MTEC retain the ability to differentiate and develop a pseudostratified epithelial layer

**Figure 2:**
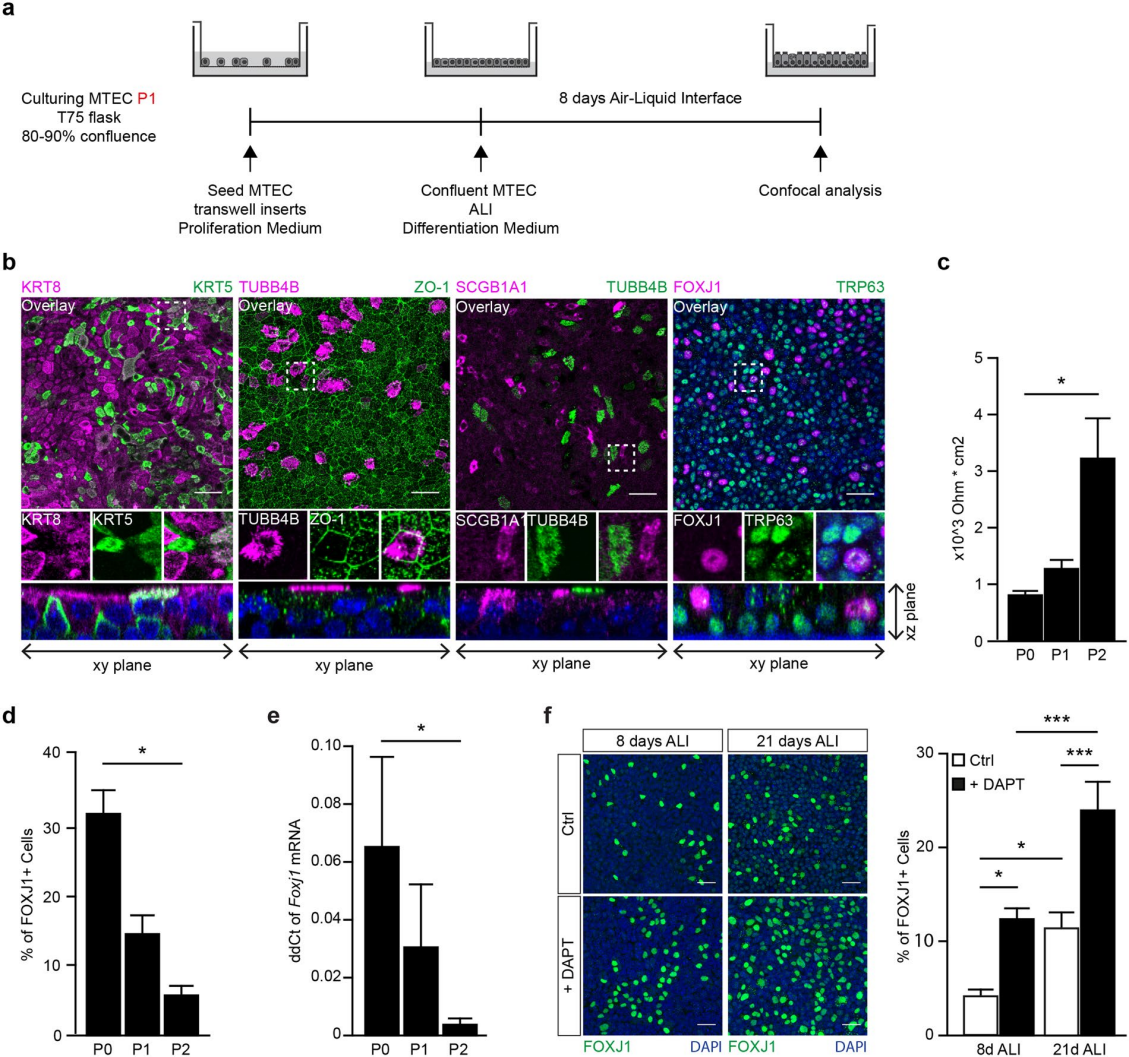
Expansion of MTEC *in vitro* leads to decreased ciliary and club cell differentiation. (**a**) Schematic representation of culture protocol. (**b**) Co-staining of different epithelial markers on MTEC passage 2 after 8 days of air liquid interface (ALI) culture. From left to right, inserts were stained with basal cell marker KRT5 and luminal marker KRT8, cilia cell TUBB4B with tight junction protein ZO-1, secretory club cell marker SCGB1A1 with cilia marker TUBB4B and the last panel shows TRP63 positive basal cells with ciliated cell marker FOXJ1. Nuclei are stained with DAPI (blue). Scale bar, 30 µm. (**c**) Resistance measurements of MTEC after different passages and 8 days of ALI (mean ± SEM). *p < 0.05 by one-way ANOVA (n = 3). (**d**) Quantification of the percentage of FOXJ1 positive ciliated cells after 8 days of ALI comparing MTEC in passage (P) 0, 1 and 2 (mean ± SEM). *p < 0.05 by one-way ANOVA (n = 3). (**e**) qRT-PCR analysis of *Foxj1* in MTEC in passage (P) 0, 1 and 2 (mean ± SEM). *p < 0.05 by one-way ANOVA (n = 4). (**f**) Representative images and quantification of ciliated cells (FOXJ1) after 8 days or 21 days ALI and with or without the presence of DAPT during differentiation (mean ± SEM). *p < 0.05, ***p < 0.001 by two-way ANOVA (n = 6). Scale bar, 30 µm.

Differentiation into a pseudostratified epithelial layer is essential when using MTEC cultures to mimic *in vivo* airway epithelium. To evaluate whether MTEC that were expanded in KSFM expansion medium retain the ability to form a pseudostratified epithelial layer *in vitro*, MTEC were isolated, cultured submerged in KSFM expansion medium. Subsequently, MTEC were cultured on inserts in MTEC proliferation medium until confluent, followed by differentiation at ALI in differentiation medium (Fig. 2a). Polarization of the pseudostratified epithelial layer in an apical and basal side was evaluated using keratin 8 (KRT8) and keratin 5 (KRT5), which mark the luminal and basal cell layer respectively. Cellular differentiation was analyzed by staining for specialized cell types, including ciliated cells using forkhead box 1 (FOXJ1) and beta-tubulin IV (TUBB4B), club cells using secretoglobin, family 1A, member 1 (SCGB1A1) and basal cells using transformation-related protein 63 (TRP63). Additionally, zona occludens 1 (ZO-1)-specific staining indicated epithelial tight junction formation, an important feature of epithelial cells to maintain a tight barrier function. After 8 days of differentiation at ALI we observed stratification of the epithelial cell layer, indicated by KRT5 expression in the basal cell layer and KRT8 expression in the luminal cell layer, similar to ALI-MTEC cultures of P0 (Figs 2b and S1). Although most of the cells were single positive for either KRT5 or KRT8, some cells were KRT5/KRT8 double positive, indicating the presence of basal luminal precursors which are cells transitioning from a basal to a luminal cell phenotype^20^. Expanded MTEC developed tight junctions when cultured at ALI (Fig. 2b). Additionally, ciliated and secretory cells were detected after 8 days of differentiation at ALI (Fig. 2b). Epithelial polarization of expanded MTEC cultured at ALI was similar to the *in vivo* tracheal epithelium with TRP63 positive basal cells located at the basolateral side and the FOXJ1 positive ciliated cells at the apical side (Fig. 2b XZ plane, Fig. S1). Taken together, these data show that expanded MTEC retain the ability to develop a pseudostratified epithelial layer, mimicking the *in vivo* pseudostratified epithelium. We next compared the epithelial differentiation potential between P0, P1 and P2 MTEC by assessing ciliated cells and barrier development after 8 days ALI differentiation. Whereas the P2 ALI-MTEC were able to develop ciliated cells, the percentage of FOXJ1 positive cells was lower compared to passages P0 and P1 after 8 days of ALI differentiation (Figs 2d,e and S2). Barrier function was assessed using trans-epithelial resistance measurement, whereby the reduction in FOXJ1 positive cells was accompanied by a significant increase in barrier development between P0 and P2 ALI MTEC (Fig. 2c–e). In line with our immunofluorescence imaging, we observed a significant decrease in *Foxj1* mRNA expression between P0 and P2 ALI MTEC (Fig. 2e). In addition to a decrease in FOXJ1 positive cells, we also observed a decrease in club cells as indicated by decrease in SCGB1A1 and SCGB3A2 positive cells between P0 and P2 ALI MTEC as detected by immunofluorescence staining and mRNA expression (Fig. S2c and d). Next we investigated whether the passaged MTEC could be treated in such a way that they develop ciliated cells in P2 ALI MTEC. Previously, it was shown that the presence of DAPT during differentiation increases the development of ciliated cells^11,21^. To this end, we differentiated expanded MTEC in the presence of DAPT during ALI. Aside from the expected decrease of club cells^18^, we found an increased number of FOXJ1 positive ciliated cells compared to MTEC that were differentiated at ALI without DAPT Moreover, increasing the duration of ALI differentiation also increased the number of FOXJ1 positive cells (Fig. 2f). Overall, expanded MTEC cultured at ALI retain their ability to develop a pseudostratified epithelial layer. The differentiation of expanded MTEC appeared to be altered as indicated by a decrease in ciliated cells compared to P0. However, addition of DAPT during differentiation and increasing ALI duration increases the number of ciliated cells comparable to the numbers as observed in P0.

### MTEC differentiation can be modulated using IL-13 to induce goblet cell metaplasia

**Figure 3:**
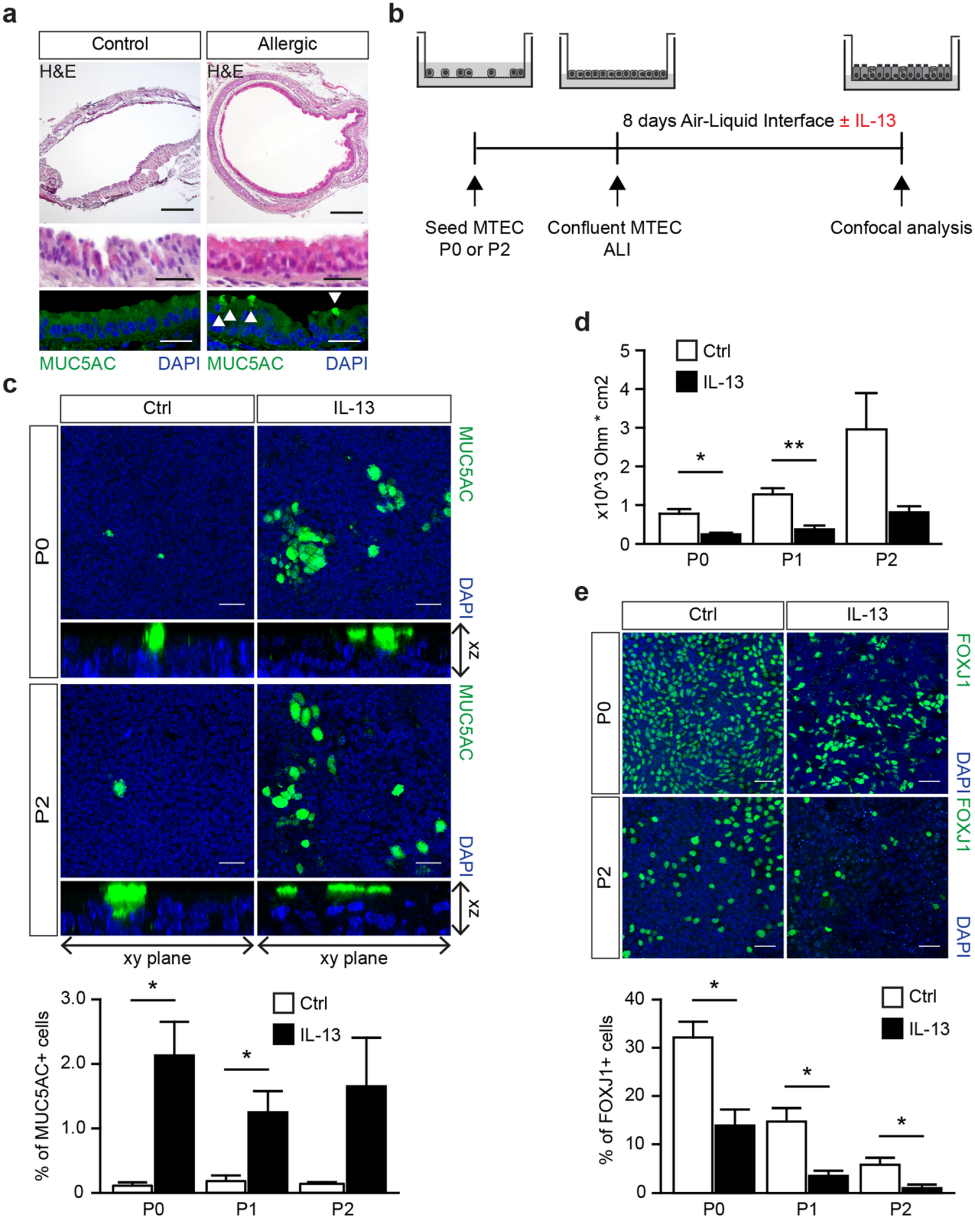
IL-13 exposure during MTEC differentiation induces goblet cells. (**a**) Hematoxylin and eosin on tracheal sections of control or house dust mite (HDM) treated mice. Immunofluorescence staining with Mucin 5AC (MUC5AC) shows the presence of goblet cells. MUC5AC expressing cells are indicated by the white arrows. Scale bar, 200 µm and 30 µm. (**b**) A schematic representation of the culture protocol. (**c**) Immunofluorescence staining of MUC5AC of MTEC passage 0 and 2 after 8 days of ALI culture with or without IL-13. Scale bar, 30 µM. Graph shows the percentage of MUC5AC expressing goblet cells in culture (mean ± SEM). *p < 0.05 by Student two-tailed t-test (n = 3). (**d**) Resistance measurements after 8 days of ALI culture with or without IL-13 (5 ng/ml) treatment (mean ± SEM). *p < 0.05 by Student two-tailed t-test (n = 3). Scale bar: 30 µm. (**e**) Immunofluorescence staining of FOXJ1 positive ciliated cells in a passage 0 (P0) and 2 (P2) after 8 days of ALI culture with or without IL-13 (5 ng/ml). Scale bar, 30 µm. Graph show the percentage of FOXJ1 expressing ciliated cells in culture (mean ± SEM). *p < 0.05 by Student two-tailed t-test (n = 3).

Expanded MTEC cultured at ALI retained the ability to develop a pseudostratified epithelial layer. Next, we evaluated whether MTEC differentiation could be modulated using IL-13, which is important in the development of goblet cell metaplasia in mouse models of allergic airway inflammation^22,23^. We stained trachea of mice that were sensitized and exposed to house dust mite (HDM) allergen to induce allergic airways inflammation for the presence of goblet cells, and found these to be increased compared to placebo-treated mice (Fig. 3a). Previously, IL-13 has been shown to induce goblet cell metaplasia *in vitro* using ALI cultures of airway epithelial cells^15,24–26^. We exposed non-expanded (P0) and expanded (P1, P2) MTEC with DAPT to IL-13 during ALI culture and compared IL-13-induced MUC5AC expression patterns. Goblet cells are scarce in MTEC after 8 days of differentiation (Fig. 3c). However, we found that IL-13-exposed ALI MTEC cultures displayed increased numbers of goblet cells and there was no difference in the increase in goblet cells between P0 and P2 (Figs 3c and S3). Furthermore, IL-13 exposure resulted in a reduced number of ciliated cells in both ALI cultures with freshly isolated MTEC or with expanded MTEC (Fig. 3e). Finally, IL-13 decreases epithelial barrier function *in vitro*^27,28^, which is in line with our results showing a similar reduction in barrier function following IL-13 exposure in expanded MTEC and isolated MTEC cultured directly onto inserts (Fig. 3d). Taken together our results indicate that expanding MTEC *in vitro* does not affect their ability to induce a physiologic response to IL-13 exposure, suggesting that this model could be an attractive alternative to evaluate new therapeutic compounds and their effects on airway epithelial cells *in vitro*.

### Intrinsic changes of the basal cell population result in decreased differentiation potential

**Figure 4:**
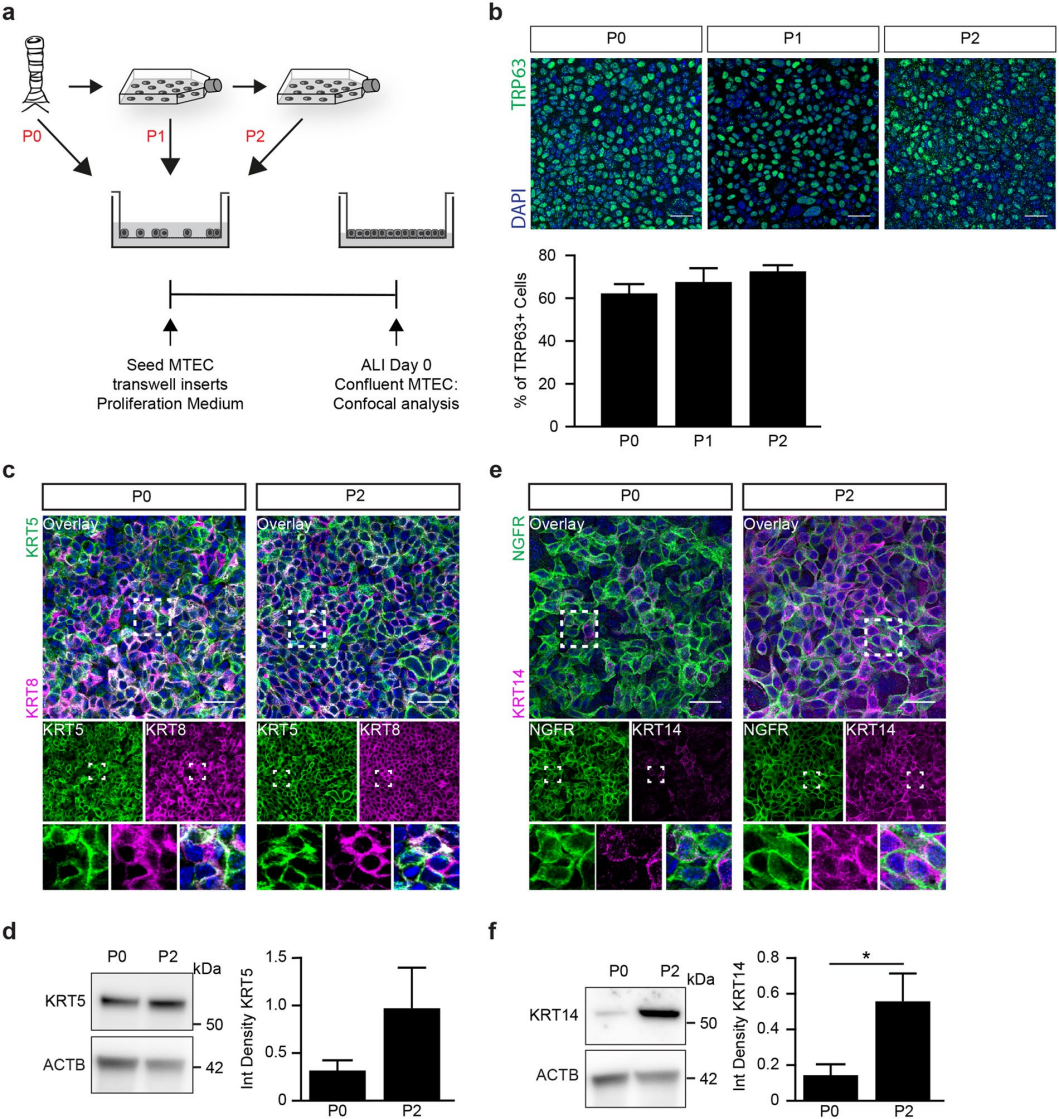
The population of TRP63 positive basal cells change during expansion. (**a**) Schematic representation of culture protocol. (**b**) Representative images of the number of TRP63 positive basal cells at ALI day 0. Scale bar, 30 µM. Graph shows the percentage of TRP63 positive basal cells at day 0 of ALI culture in a passage 0 (P0), passage 1 (P1) and passage 2 (P2) (mean ± SEM). One-way ANOVA (n = 3). (**c**) Immunofluorescence of basal cell marker KRT5, and luminal cell maker KRT8 at day 0 of ALI. The overlay picture and the one-channel pictures are shown. The boxes indicate the area of which an enlarged image is presented. Scale bar: 30 µm. (**d**) Western blot analysis of KRT5 at day 0 of ALI in a passage (P) 0 and 2. Beta-actin (ACTB) is used as loading control. *p < 0.05 by Student two-tailed t-test (n = 7). (**e**) Immunofluorescence of basal cell marker, P75-nerve growth factor (NGFR) and basal cell maker keratin 14 (KRT14) at day 0 of ALI. Scale bar: 30 µm. (**f**) Western blot analysis of KRT14 at day 0 of ALI in a passage (P) 0 and 2. Beta-actin (ACTB) is used as loading control. *p < 0.05 by Student two-tailed t-test (n = 9).

Expanding MTEC before ALI differentiation resulted in reduced numbers of ciliated cells, suggesting an altered differentiation potential of the basal cell population. The basal cell population consists of various subtypes with different functionality that can be identified by the presence of different basal cell markers^5,7^. To evaluate whether the altered differentiation potential could be explained by a change in basal cell composition at baseline, we first examined the TRP63 positive basal cell population at day 0 of ALI differentiation for P0, P1 and P2 (Fig. 4a). No significant differences were detected in the percentage of TRP63 positive basal cells between P0, P1 and P2 (Fig. 4b). This indicates that a difference in the number of TRP63 positive basal cells is not the cause of an altered differentiation potential, suggesting that the identity of the TRP63 positive basal cell is changed. We therefore next evaluated the expression patterns of other previously published basal cell markers. KRT8 and KRT5 were already used to show stratification of the MTEC cultured at ALI (Fig. 2). As indicated, KRT5 + KRT8 double positive cells mark a population of basal luminal precursor cells. Another marker used to distinguish basal cells is P75-nerve growth factor receptor (NGFR), which is enriched in murine tracheal basal cells^6^. We observed no overt differences in KRT5, KRT8 and NGFR positive basal cells in P2 compared with P0 (Figs 4c,d,e and S4).

A subpopulation of keratin 14 (KRT14) positive basal cells is reported to be present in a small subset of basal cells, but is highly increased after injury and serves as a progenitor for ciliated and secretory cells^29,30^. We investigated the presence of KRT14 positive basal cells in P0 and P2 MTEC on inserts at day 0 of ALI. The KRT14 positive basal cell fraction showed a marked increase in P2 compared to P0 (Fig. 4e and f). Overall, our data suggests that Notch signaling is involved in the maintenance of the basal cell population and that expanding MTEC during Notch signaling inhibition increases the KRT14 positive basal cell fraction without affecting the other investigated basal cell populations.

## Discussion

Using a combination of Notch signaling and Rho-associated kinase (ROCK) inhibition and specialized media, we were able to subculture MTEC while preserving the mucociliary differentiation potential when cultured at ALI. This protocol provides a much more efficient way to culture MTEC, increasing the number of experiments that can be performed with two mouse tracheas, with approximately 42.5 million cells after passaging, which is in stark contrast to 200 000 cells if MTEC are plated on transwell inserts directly after isolation. Our culture method will contribute to a reduction in the number of animals used, in line with the 3R principle, and also has the potential to decrease technical and biological variation between experiments. Furthermore, a reduction in the number of animals needed is relevant when MTEC are isolated from difficult-to-breed mouse strains. To increase the efficient use of mouse trachea for MTEC culture, we have used KSFM medium to prevent outgrowth of fibroblasts (a common problem in MTEC cultures) supplemented with the RhoA-kinase inhibitor Y-27632 and the γ-secretase inhibitor and indirect Notch signaling inhibitor DAPT. Additionally, our results indicate that expanding MTEC *in vitro* results in altered stemness of the basal cell population which is accompanied by a shift of a KRT14 negative basal cell population observed at P0 towards a KRT14 positive basal cell population at P2. Further investigation is required to evaluate the contribution of the KRT14 positive basal cell population towards the altered stemness of the basal cell fraction.

MTEC have previously been shown to have the ability to grow in submerged culture. However, the ability of the MTEC to differentiate at ALI was not explored^31^. More recently, SMAD signaling inhibition was shown to promote airway basal stem cell expansion *in vitro* from multiple species with subsequent ALI differentiation^32^. Although this study contributed significantly to the *in vitro* research field, the authors did not elaborate on cell culture techniques and growth media used. We used the SMAD signaling inhibitors described by Mou and colleagues in combination with KSFM medium, but observed that MTEC could not be expanded under these growth conditions (results not shown). In the present study, we have used KSFM to expand MTEC *in vitro* prior to culturing on transwell inserts. KSFM has previously been used successfully to expand primary human bronchial epithelial cells^15,16^. In addition, KSFM has the advantage of preventing outgrowth of fibroblasts, which is likely attributed to the low calcium levels, thus impairing fibroblast migration and proliferation^33–35^. An important advantage of the use of KSFM is therefore that it circumvents the need of culturing the isolated cell suspension for 4 to 5 hours on culture dishes to remove fibroblasts by adherence. This is important, since maintaining epithelial cells for several hours in suspension prevents them from adhering to a matrix, which may result in anoikis^36^. Our results indicate that MTEC do proliferate in KSFM, but the morphology, survival and expansion rates drastically improved when a ROCK inhibitor in combination with a Notch signaling inhibitor was used. Following expansion of MTEC in KSFM, we allowed MTEC to grow and differentiate on transwell inserts as previously described^14,17,37^.

Expanding the progenitor basal cell population is essential for subculturing MTEC *in vitro*. ROCK inhibition is frequently used in embryonic stem cell cultures, induced pluripotent stem cells and some tissue-specific stem cell populations. More specifically, the ROCK inhibitor Y27632 has previously been shown to improve proliferation rates of human and mouse tracheal epithelial cells without affecting subsequent ALI differentiation^17^. Feeder layers or conditional medium from feeder layers has proven effective in expanding airway epithelial cells^38^. However, maintaining feeder layers adds significantly to the workload and exact mechanisms through which the feeder layers improve proliferation remain unknown. Therefore, using a defined culture medium without the need of feeder layers is preferred and is likely to result in increased consistence of the culture system.

A mechanism that has been suggested to underlie the ability of ROCK inhibitors to improve cell proliferation is the inhibition of downstream Notch signaling^39,40^. Whereas Y27632 improved MTEC proliferation rates in our experiments, cell morphology and survival suggested the presence of cell contact-induced impairment of cell proliferation. Cell-cell communication and related inhibition of proliferation has previously been attributed to Notch signaling^41^. Notch signaling has previously been shown to be involved in basal epithelial cell proliferation and differentiation both *in vitro* and *in vivo*^10,11,18,21,41,42^. We therefore used DAPT, a γ-secretase inhibitor and indirect Notch signaling inhibitor, in combination with Y27632 in KSFM to expand MTEC *in vitro*. Our data shows that inhibiting Notch signaling is an effective way to expand the basal cell population.

Differentiation of the basal cell population into a pseudostratified epithelial layer containing secretory and ciliated cells is an important feature of the airway epithelium. We have shown that MTEC expanded to passage 2 retain the ability to differentiate into a pseudostratified epithelial layer. To this end, we used KRT5 and KRT8 staining to discriminate between basal and luminal precursor populations. Some cells show the presence of both KRT5 and KRT8 which may mark basal cells differentiating towards a luminal precursor cell^11,20^. Furthermore, the pseudostratified epithelium of the trachea contains various cell types including basal, ciliated and secretory cells. Using FOXJ1 and TUBB4B staining for ciliated cells and SCGB1A1 and SCGB3A2 staining for secretory cells in addition to qRT-PCR, we showed the presence of ciliated and club cells in expanded MTEC. However, the expanded MTEC had reduced numbers of ciliated and secretory cells present after 8 days of ALI, despite the unchanged KRT8 positive luminal fraction in P2 compared to P0. This suggests that the cells may require more time or an additional trigger for efficient end-stage differentiation. This hypothesis is strengthened by the observed increase in ciliated cells by extending the duration of ALI. This was further increased by adding the Notch inhibitor DAPT during ALI, which is in line with previous studies showing that inhibition of Notch signaling may drive epithelial cells towards ciliated cells^21,43^. Collectively, these findings indicate that after expansion in culture, basal cells retain the ability to develop a pseudostratified epithelial layer following ALI differentiation, but with an altered differentiation capacity, likely resulting from altered stemness of the basal cell population. Additional triggers generated by extended culture and Notch inhibition, are required to achieve more efficient mucociliary differentiation.

The ability to adapt to a changing environment to various stimuli is an important feature of airway epithelial cells. Furthermore, appropriate *in vitro* epithelial culture models should recapitulate the response to disease-specific stimuli observed in patients and/or animal models of disease. To this end, we used IL-13 exposure and assessment of subsequent goblet cell development of expanded MTEC cultured at ALI to mimic the *in vivo* allergic airways inflammation. IL-13 is a T helper 2 cytokine known to induce goblet cell metaplasia *in vivo* and *in vitro*^22,23,25,44,45^. IL-13 exposure during differentiation induced MUC5AC positive goblet cells in both expanded MTEC and in freshly isolated MTEC that were cultured directly onto inserts. The presence of IL-13 during differentiation at the ALI resulted in an increased number of goblet cells, likely resulting from basal cells differentiating towards goblet cells rather than ciliated cells. Alternatively, the increased number of goblet cells may be resulting from IL-13 induced trans-differentiation of ciliated cells towards goblet cells, resulting in fewer ciliated cells^26,45,46^. Moreover, IL-13 induced a reduction in barrier function in both expanded MTEC and isolated MTEC that had been cultured directly onto transwell inserts, which is in line with previous publications showing that IL-13 reduces barrier function *in vitro*^27,28^. Overall, expanded MTEC showed a robust response following IL-13 exposure including decreased barrier function and the development of goblet cells, indicating that expanding MTEC *in vitro* using a Notch signaling inhibitor does not affect their ability to respond to IL-13. The observation that goblet cell differentiation induced by IL-13 exposure is similar in P0 and P2, suggests that the basal cells retain the ability to differentiate into goblet cells.

Basal cells are the main progenitor cells of the conducting airways. They have the proficiency to self-renew and differentiate into various cell types found in a pseudostratified epithelial layer including ciliated and secretory cells. Various cellular markers have been described to distinguish basal cells in the airway epithelium. Furthermore, basal cells can be separated in various subsets depending on the combination of cellular markers present^5,7^. KRT5, TRP63 and NGFR are commonly used markers to delineate basal cell populations. In contrast, only a small fraction of basal cells is positive for KRT14 at baseline. Our results indicated that expansion significantly induced a KRT14 positive basal cell population in P2 compared to P0, whereas the other investigated basal cell populations remained unaltered between passages. Together these data suggest that expanding basal cells *in vitro* can alter the composition of the basal cell population, which may explain why we see a reduced number of ciliated cells in expanded MTEC compared to isolated MTEC cultured directly onto inserts. Whether this alteration of the basal cell population results from the KRT14 negative basal cell population turning KRT14 positive or that the KRT14 positive basal cell population has an increased capacity to expand *in vitro* compared to the KRT14 negative basal cell population remains to be further investigated. However, KRT14 positive basal cells are still able to differentiate in ciliated cells and goblet cells when exposed to additional triggers, respectively DAPT and IL-13, is a similar way as KRT14 negative basal cells. More research is needed to investigate the role of KRT14 positive basal cells and their ability to differentiate.

In conclusion, we developed a clearly defined culture method that allows ALI differentiation of MTEC into a pseudostratified epithelial layer following *in vitro* expansion. Expanding MTEC *in vitro* did result in decreased differentiation of basal cells to ciliated cells, likely resulting in part from an altered composition of the basal cell population as indicated by an increase in KRT14 positive basal cells. Despite the altered basal cell population, the Th2 cytokine IL-13 was still able to redirect epithelial differentiation towards a phenotype observed in allergic airways inflammation. Furthermore, this culture method may be useful to study repair following injury combined with *in vitro* lineage tracing if MTEC from transgenic animals are used. Also, this new culture method will contribute to a reduction in animals needed for experimentation, in line with the principles of the 3R’s (Replacement, Reduction and Refinement).

## Methods

### Culture media and supplements

A detailed overview of all culture media and supplements can be found in Supplemental Table 1. “Ham’s F12” is defined as Ham´s F12 (Gibco, Bleiswijk, The Netherlands) supplemented with 100 U/ml penicillin and 100 µg/ml streptomycin (Lonza, Verviers, Belgium). “MTEC basic” medium is defined as DMEM/F12 (Gibco) supplemented with 100 U/ml penicillin, 100 µg/ml streptomycin and 0.03% (w/v) NaHCO_3_ (Gibco). “KSFM expansion medium” is defined as KSFM (Gibco) supplemented with 1% penicillin-streptomycin, 1 µM isoproterenol (Sigma-Aldrich, St. Louis, MO, USA), 0.03 mg/ml bovine pituitary extract (Gibco), 25 ng/ml murine epidermal growth factor (Peprotech, Rocky Hill, NJ, USA) and 10 µM Y-27632 hydrochloride (Cayman Chemical, Ann Arbor, MI, USA). “MTEC proliferation medium” is defined as MTEC basic supplemented with 5% (v/v) Fetal Bovine Serum (FBS), 1.5 mM L-glutamine (Lonza), 1 × (v/v) Insulin-Transferrin-Selenium (Gibco), 0.1 µg/ml cholera toxin (Sigma-Aldrich), 25 ng/ml murine epidermal growth factor, 0.03 mg/ml bovine pituitary extract, 0.05 µM retinoic acid (Sigma-Aldrich) and 10 µM Y-27632 hydrochloride. “MTEC differentiation medium” is defined as MTEC basic supplemented with 0.1% (w/v) bovine serum albumin (BSA) (Gibco), 1.5 mM L-glutamine, 1% (v/v) Insulin-Transferrin-Selenium, 25 ng/ml cholera toxin, 5 ng/ml murine epidermal growth factor, 0.03 mg/ml bovine pituitary extract and 0.05 µM retinoic acid. Retinoic acid and Y-27632 hydrochloride stocks were prepared and stored at −80 °C and −20 °C respectively, and were supplemented fresh for cell culture usage and used within the same day. Culture media containing retinoic acid were protected from light.

### Mouse tracheal epithelial cells (MTEC) isolation and culture

All animal experimental protocols were approved by the animal welfare committee of the veterinary authorities of the Leiden University Medical Center or the Erasmus Medical Center. Wild-type C57Bl/6 mice were used for isolating murine tracheas. MTEC were isolated as described previously^14^. After isolation, MTEC were resuspended in MTEC basic containing 10% FBS for direct culture on transwell inserts, or in KSFM for further passage and expansion of MTEC.

In experiments in which MTEC were grown on transwell inserts directly after isolation without passaging of the cells in KSFM expansion medium, the cells were first deprived of fibroblasts by incubating the cells on Primaria plates (Corning Costar, Cambridge, MA, USA) for 5 h at 37 °C with 5% CO_2_. Non-adherent cells were collected and centrifuged at 390 × g for 10 min at room temperature. Cells were resuspended in MTEC proliferation medium, plated at 8 × 10^4^ cells/cm^2^ on transwell inserts (0.4 µm pore size; Corning Costar) and cultured submerged until confluent at 37 °C with 5% CO_2_. MTEC proliferation medium was refreshed every two to three days.

For MTEC subculture experiments, trachea-derived cells were collected in KSFM expansion medium and plated in T75 flasks at 5 × 10^3^ cells/cm^2^. The adherence step using Primaria plates to remove fibroblasts was omitted when using KSFM, since this medium is formulated by the provider to prevent fibroblast growth. KSFM expansion medium was supplemented with DAPT (Sigma-Aldrich), a Notch signaling inhibitor, according to the experimental design described in the results section. At 80–90% confluence, MTEC were passaged using 0.25% (w/v) trypsin (Gibco) and 2.7 mM EDTA (Sigma-Aldrich) in Cell Dissociation Solution Non-enzymatic 1x (Sigma-Aldrich). MTEC were plated at 5 × 10^3^ cells/cm^2^ in KSFM. Passage 1 or passage 2 MTEC were used for further experiments. For culturing onto transwell inserts, cells were dissociated with Cell Dissociation Solution (Sigma-Aldrich) supplemented with 0.25% trypsin and 2.7 mM EDTA at 37 °C for 15 min, centrifuged at 390 g for 10 min at room temperature and resuspended in MTEC proliferation medium. MTEC were plated on transwell inserts at 8 × 10^4^ cells/cm^2^ and cultured in MTEC proliferation medium until confluent at 37 °C with 5% CO_2_. MTEC proliferation medium was refreshed every two to three days. A schematic overview of the expansion method followed is given in Fig. 1.

After MTEC cultured on transwell inserts had grown to full confluence, apical medium was removed to achieve air-liquid interface (ALI) culture conditions. After the start of culture at ALI, the apical surface was washed with warm PBS and the basal medium was refreshed using MTEC differentiation medium every two to three days.

For experimental exposures, ALI-MTEC were exposed to either 5 ng/ml murine IL-13 (Peprotech) or 5 µM DAPT, added in the basolateral chamber of the transwell insert for the indicated duration.

### RNA extraction, cDNA synthesis and Quantitative RT-PCR analysis

Cells were collected in PBS from the insert using cell-scrapers on ice. After centrifugation at 4 °C, 5000 rpm for 5 min, the cell pellet was lysed using TRIzol^TM^ reagent (Life Technologies, Rockville, MD, USA). RNA extraction was performed according to the TRIzol^TM^ Reagent protocol. RNA concentrations were measured using Nanodrop (Thermofisher Scientific). First strand cDNA synthesis was synthesized using MLV Reverse transcriptase (Sigma, M1302) with Oligo(dT) primers (self-designed: 23xT + 1 A, 23xT + 1 C and 23xT + 1 G). Quantitative RT-PCR analysis was performed with 0.5 µl of cDNA per reaction, Platinum Taq polymerase (Invitrogen, 18038042) and SybrGreen (Sigma, S9430). The primer combinations for the qRT-PCR are listed in Supplemental Table 3. Relative gene expression was calculated using the ∆∆CT method relative to GAPDH control.

### Allergic airway inflammation in mice

To induce allergic airway inflammation in mice, 8 to 12 weeks old female C57BL/6 mice were sensitized by intranasal instillation of 1 µg *Dermatophagoides pteronyssinus* extract (house dust mite extract (HDM)) in 50 µl PBS, followed by 5 daily intranasal challenges with 10 µg HDM in 50 µl PBS or PBS starting at 7 days after sensitization. Mice were sacrificed 2 days after the last challenge. Trachea were removed and fixed in 4% paraformaldehyde (PFA) (Sigma-Aldrich) and stained for confocal analysis as described below.

### Immunofluorescence of ALI-MTEC culture

MTEC on inserts were rinsed with PBS followed by fixation with 4% (w/v) paraformaldehyde (PFA) for 10 min at room temperature. After fixation cells were washed 3 times with PBS/0.2% (v/v) Triton-X100 (Sigma-Aldrich) to remove residual mucus from the apical surface. Non-specific binding sites were blocked with 5% (v/v) donkey serum (EMD Millipore, Billerica, MA, USA), 1% (w/v) BSA and 0.2% (v/v) Triton-X100 in PBS for 30 min at room temperature, followed by incubation with primary antibodies in blocking buffer at 4 °C overnight (Supplemental Table 2). The next day, MTEC were washed three times with PBS/0.02% Triton X-100 and incubated for 2 h at room temperature with Alexa Fluor labeled secondary antibodies (Jackson ImmunoResearch, West Grove, PA, USA) in blocking buffer (Supplemental Table 2). After three washing steps with PBS/0.02% (v/v) Triton-X100 and one washing step with PBS, the cells were mounted with Vectashield hardset containing DAPI (Vector Laboratories, Burlingame, CA, USA). Images were acquired using a Leica SP5 confocal microscope. Cell counting was performed using ImageJ and 10–15 areas from each insert were analyzed for each replicate.

### Immunofluorescence of tracheal sections

Mouse tracheas were fixed with 4% PFA overnight at 4 °C, washed with PBS and embedded in paraffin for sectioning. Tracheas were sectioned (5 µm) and deparaffinized, rehydrated and subjected to antigen retrieval in Tris-EDTA (pH 9.0) at 600 W for 15 min. After blocking with PBS plus 5% donkey serum and 0.05% Tween-20, sections were incubated with primary antibodies in blocking buffer overnight at 4 °C (Supplemental Table 2). The next day, sections were washed with PBS/0.05% Tween (3x, 5 min). Alexa Fluor labeled secondary antibodies were used in a 1:500 dilution in blocking buffer and sections were incubated for 2 h at room temperature. After incubation, sections were washed (3x, 5 min) with PBS/0.05% Tween-20 followed by one final wash with PBS only. Sections were mounted with Vectashield hardset containing DAPI (Vector Laboratories) and confocal images were obtained using a Leica SP5 confocal microscope.

### Trans-epithelial electrical resistance (TEER)

TEER values were measured with an STX2 electrode (World Precision Instruments, Berlin, Germany) connected to a Millicell ERS voltohmmeter (World Precision Instruments). Prior to measurement, 700 µl prewarmed PBS was added apically to the ALI-MTEC and incubated at room temperature for 10 min. Resistance measurements were corrected for the surface of the transwell inserts and expressed as Ohm * cm^2^.

### Western blot analysis

Cells were washed twice with PBS, scraped of the insert in PBS and pelleted. Cells were lysed in Carin lysis buffer containing 20 mM Tris-HCl pH 8.0, 137 mM NaCl, 10 mM EDTA, 10% glycerol and 1% NP-40 and incubated on ice for 15 min. Complete protease inhibitors (Roche) was freshly added each time to the lysis buffer. Samples were centrifuged for 5 min at 18,711 g in an Eppendorf5417R at 4 °C. Pellets were discarded and supernatant was used for western blot analysis. Protein concentrations were determined by the Pierce® BCA Protein Assay Kit (Thermos scientific) and equal concentrations of protein were eluted in 4 × SDS sample buffer and 50 mM 1,4-dithiothreitol (DTT, sigma). Samples were boiled and loaded on a 12% SDS-polyacrylamide gel and blotted onto a PVDF membrane (Immobilon®-P transfer membrane, Millipore). The blots were blocked for 1 h in PBS containing 0.05% Tween-20 and 3% BSA at room temperature, and probed overnight with primary antibodies at 4 °C. Next day, membranes were washed three times with PBS containing 0.05% Tween-20 and incubated for 1 h with horseradish peroxidase (HRP)-conjugated secondary antibodies (DAKO) at a dilution of 1:10,000. Signal was detected with Amersham^TM^ ECL^TM^ Prime Western Blotting Detection Reagent (GE Healthcare). Blots were developed using the Amersham Typhoon imaging system (GE Healthcare).

### Statistical analysis

Data are represented as means with standard error of mean of measurements. Statistical differences between samples were assessed with a one way or two way analysis of variance (ANOVA) or unpaired t-test. Differences at P-values below 0.05 are considered significant (*p < 0.05). All statistical analyses were performed using Graphpad PRISM version 5.02.

### Data availability statement

All data generated or analyzed during this study are included in this published article (and its Supplementary Information files).

## Electronic supplementary material

Supplemental data
