# Supplementary material for: A novel method for expansion and differentiation of mouse tracheal epithelial cells in culture
Source: Sci Rep. 2018 May 9;8:7349. doi: 10.1038/s41598-018-25799-6 (PMC5943313; doi:10.1038/s41598-018-25799-6)
Supplement: Supplementary file 1 — Supplemental data [file 41598_2018_25799_MOESM1_ESM.pdf]

A novel method for expansion and differentiation of mouse tracheal epithelial cells in culture

Evelien Eenjes[1,2], Tinne C.J. Mertens[1,3,4], Marjon J. Buscop-van Kempen[2], Yolanda van Wijck[3], Christian Taube[3,5], Robbert J. Rottier[2,6] and Pieter S. Hiemstra[3,6]

[1] Authors contributed equally
[2] Department of Pediatric Surgery, Erasmus Medical Center-Sophia Children's Hospital, Rotterdam, The Netherlands
[3] Department of Pulmonology, Leiden University Medical Center, Leiden, The Netherlands
[4] Present address: Department of Biochemistry and Molecular Biology, The University of Texas Health Science Center at Houston, Houston, Texas, USA
[5] Present address: Department of Pulmonary Medicine, West German Lung Center, Essen University Hospital, Ruhrlandklinik, University Duisburg-Essen, Essen, Germany
[6] These authors share senior authorship

Supplemental data

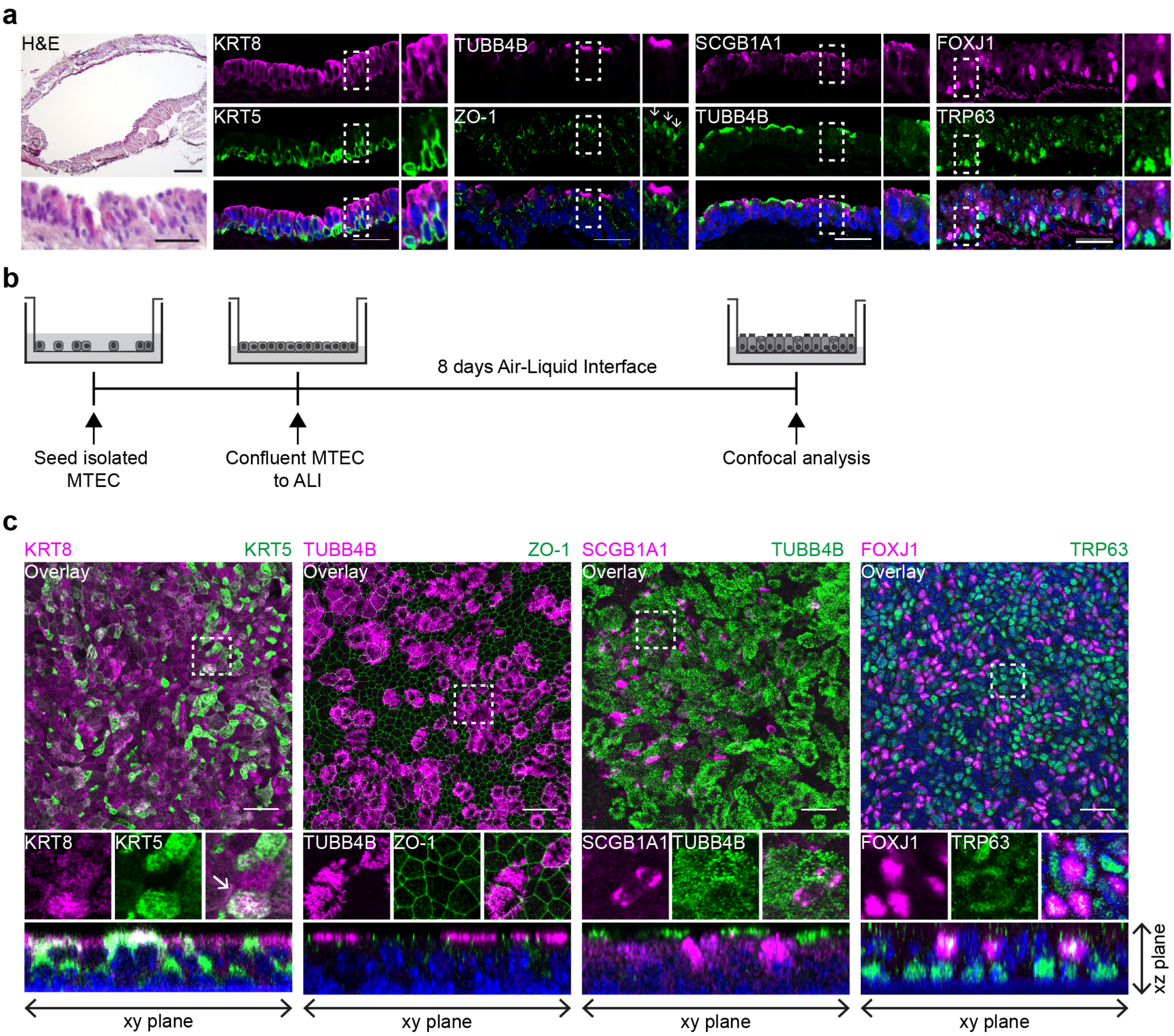

Supplemental figure 1: MTEC ALI cultures recapitulate the in vivo pseudostratified airway epithelium.

A. Hematoxylin and eosin staining of a mouse tracheal sections and immunofluorescence co-staining on tracheal sections with basal cell marker KRT5 and luminal cell marker KRT8, cilia marker TUBB4B with tight junction protein ZO-1 (indicated by the arrows), secretory club cell marker SCGB1A1 with cilia marker TUBB4B and the last panel shows TRP63 positive basal cells with ciliated cell marker FOXJ1. Nuclei are stained with DAPI (blue). Scale bar, 200 µm and 30 µm.

B. Schematic representation of ALI culture.

C. Co-staining of the different epithelial markers on MTEC ALI culture. The arrow marks a basal luminal precursor cell that is both positive for KRT5 and KRT8. Orthogonal view shows a stratified epithelium. Nuclei are stained with DAPI (blue). Scale bar, 30 µm.

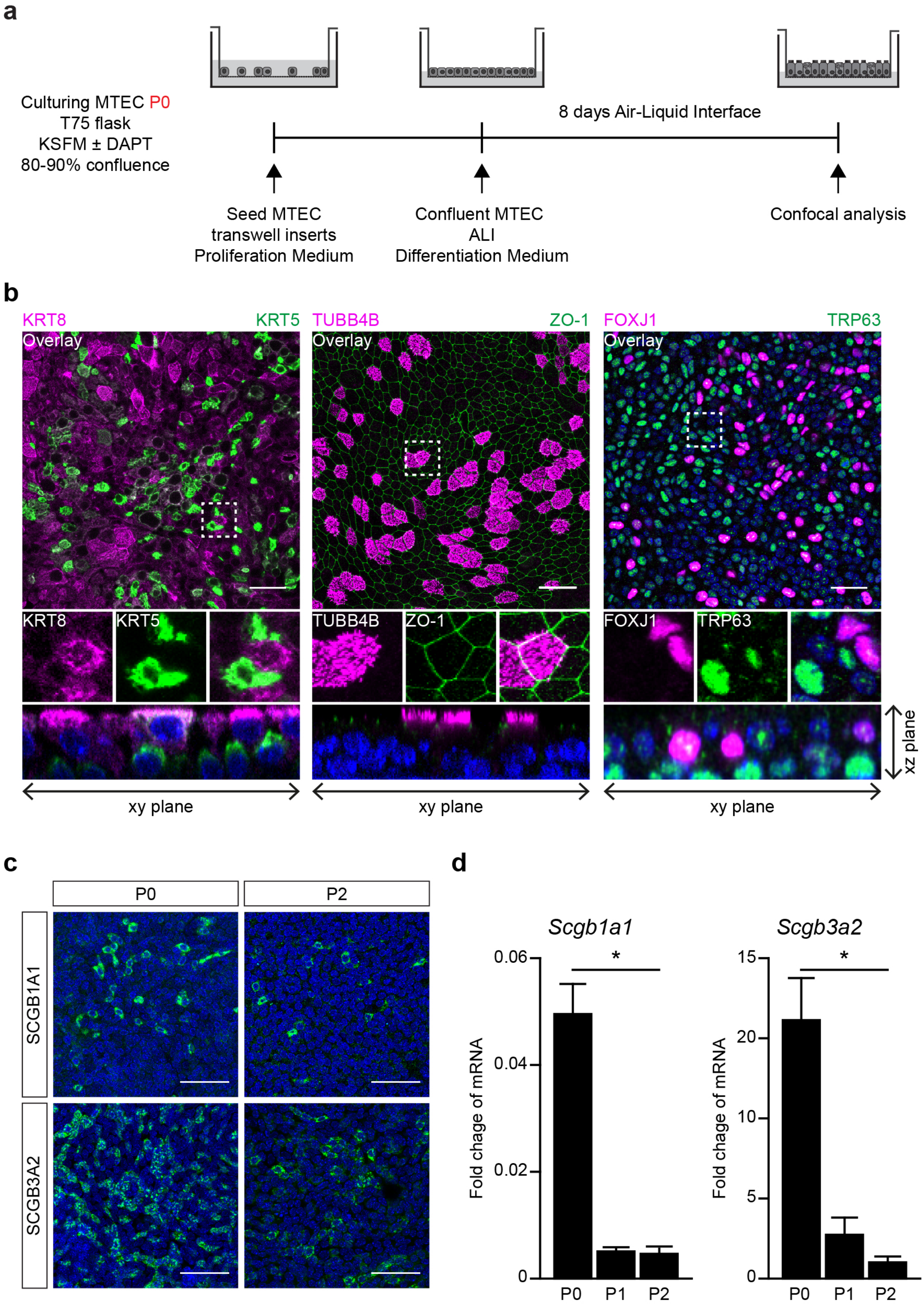

Supplemental figure 2: Representative images of P1 on inserts.
A. Schematic representation of ALI culture.
B. Co-staining of different epithelial markers on MTEC passage 1 after 8 days of Air Liquid Interface (ALI) culture. From left to right, inserts were stained with KRT5 for the basal cell layer and differentiation marker KRT8, Ciliated cell marker FOXJ1 with tight junction protein ZO-1, secretory cell marker SCGB1A1 with cilia marker TUBB4B and the last panel shows TRP63 positive basal cells with ciliated cells. Nuclei are stained with DAPI (blue). Scale bar, 30 µm.
C. Staining of SCGB1A1 an SCGB3A2 expressing secretory cells in MTEC passage (P) 0 and 2. Scale bar, 50 µm.
D. qRT-PCR of Scgb1a1 and Scgb3a2 mRNA in MTEC P0 and P2 at 8 days of ALI (mean± SEM). * $p < 0.05$ by one-way ANOVA (n=4).

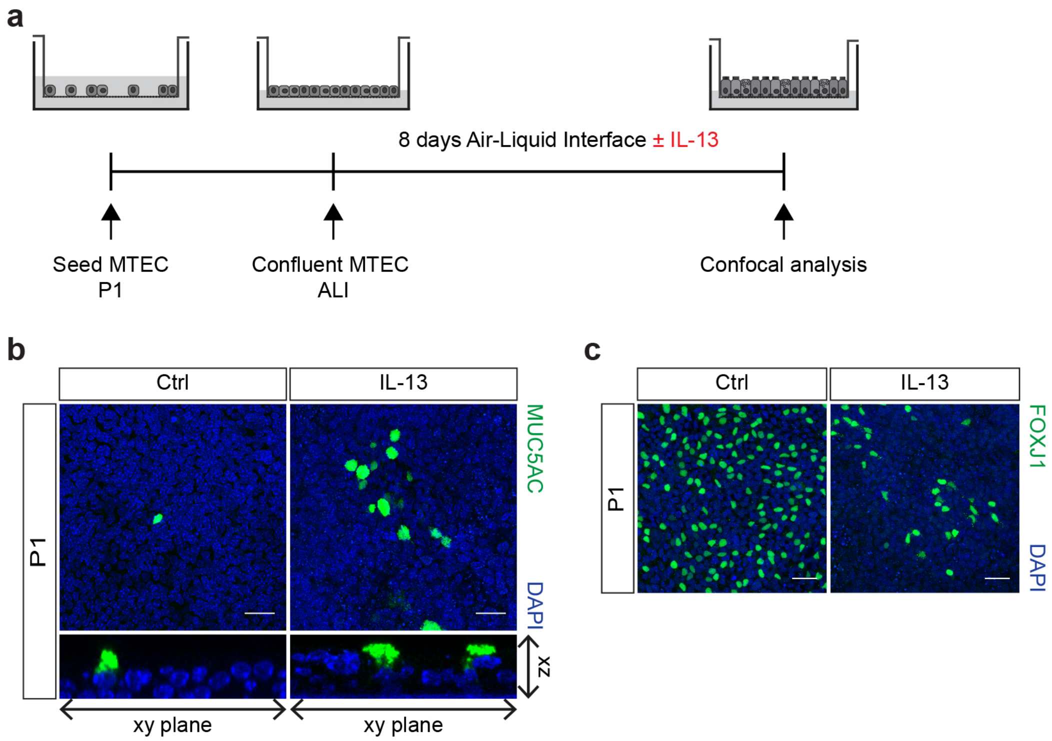

Supplemental figure 3: IL-13 treatment stimulates the formation of goblet cells in vitro.
A. Schematic representation of culture protocol.
B. Staining of Mucin 5AC (MUC5AC) expressing goblet cells in MTEC (Passage 1, P1) after 8 days of ALI with or without IL-13 (5 ng/ml). Nuclei are stained with DAPI (blue). Scale bar: 30 µM.
C. Staining of FOXJ1 positive ciliated cells after 8 days of ALI with or without IL-13 (5 ng/ml) in P1. Nuclei are stained with DAPI (blue). Scale bar: 30 µm.

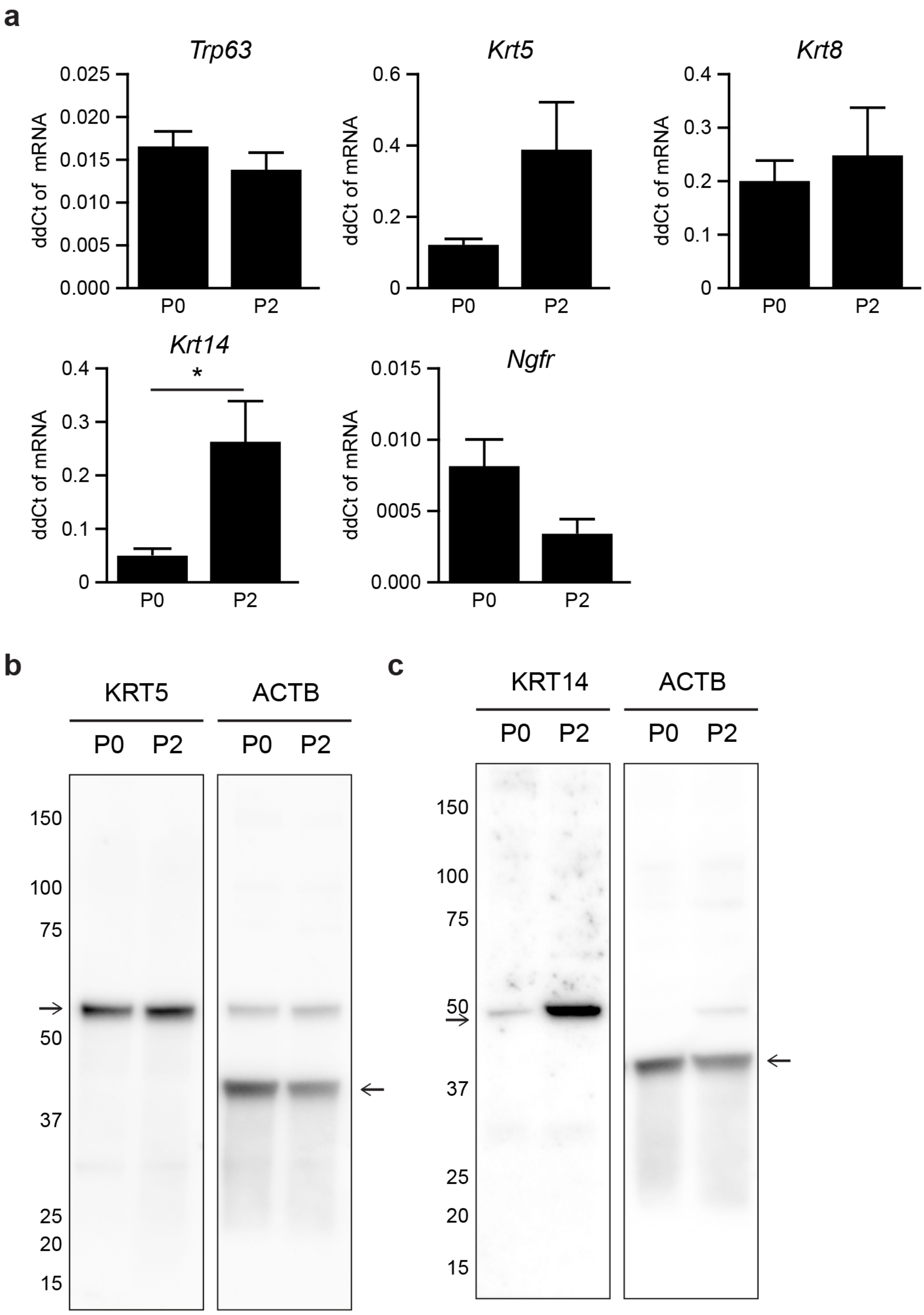

Supplemental figure 4: Western blot of Krt14 expression on ALI day 0 in P0 and P2

A. Expression of Trp63, Krt5, Krt8, Krt14 and Ngfr mRNA in MTEC Passage (P) 0 and 2 at day 0 of Air Liquid Interfase (ALI) ALI normalized to Gapdh (mean± SEM). * $p < 0.05$ by unpaired t-test (n=4).

B. Full length blots of the cropped blots of fig. 4d. Beta-Actin (ACTB) is used ad loading control and labeled on the same blot as KRT5.

C. Full length blots of the cropped blots of fig. 4f. ACTB is used ad loading control and labeled on the same blot as KRT14.

Supplemental table 1: Overview of culture media and supplements

Medium	Component	Provider<br>(catalogue number)	Final concentration
KSFM expansion medium	KSFM	Gibco (17005034)	
	Penicillin<br>Streptomycin	Lonza (DE17-602e)	100 U/ml<br>100 µg/ml
	Murine EGF	Peprotech (315-09)	0.025 µg/ml
	Bovine Pituitary Extract	Gibco (13028014)	0.03 mg/ml
	Isoproterenol	Sigma (I-6504)	1 µM
	Y-27632 (add fresh)	Cayman Chemical (10005583)	10 µM
	DAPT (add fresh)	Sigma (D5942)	5 µM
MTEC Basic (*)	DMEM/F12	Gibco (1133032)	
	Penicillin<br>Streptomycin	Lonza (DE17-602e)	100 U/ml<br>100 µg/ml
	$NaHCO_3$	Gibco (25080094)	0.03% (w/v)
MTEC proliferation medium	MTEC basic	(*)	
	L-Glutamine	Gibco (25030081)	1.5 mM
	Fetal Calf Serum	HyClone (SH30071.03)	5%
	ITS-G	Gibco (41400045)	1x
	Cholera Toxin	Sigma (C8052-0.5mg)	0.1 µg/ml
	Murine EGF	Peprotech (315-09)	0.025 µg/ml
	Bovine Pituitary Extract	Gibco (13028014)	0.03 mg/ml
	Y-27632 (add fresh)	Cayman Chemical (10005583)	10 µM
	Retinoic Acid	Sigma (R2625-50mg)	0.05 µM
MTEC differentiation medium	MTEC basic	(*)	
	L-Glutamine	Gibco (25030081)	1.5 mM
	Bovine Serum Albumin	Gibco (15260037)	0.1% (w/v)
	ITS-G	Gibco (41400-045)	1x
	Cholera Toxin	Sigma (C8052-0.5mg)	0.025 µg/ml
	Murine EGF	Peprotech (315-09)	0.005 µg/ml
	Bovine Pituitary Extract	Gibco (13028-014)	0.03 mg/ml
	Retinoic Acid	Sigma (R2625-50mg)	0.05 µM

Supplemental table 2: Antibody overview

**Primary Antibody**	**Provider (Catalogue number)**	**Dilution**
Beta-Tubulin IV	BioGenex, MU178-UC, Mouse monoclonal	1:100
SCGB1A1 (CCSP)	From Dr. Barry Stripp, Goat polyclonal	1:5000
SCGB1A1 (CCSP,<br>Uteroglobin)	Abcam, ab40873, Rabbit polyclonal	1:200
UGRP1/SCGB3A2	R&D Systems, af3465, Goat polyclonal	1:500
FOXJ1	eBioscience, 14-9965, Mouse monoclonal	1:300
KRT5	Biologend, Poly19055, Rabbit polyclonal	1:500
KRT8	DSHB, TROMA-I, Rat monoclonal	1:100
MUC5AC	Abcam, ab3649, Mouse monoclonal	1:500
TRP63	Santa Cruz, sc-8343, Rabbit polyclonal	1:50
Zona Occludens 1	Invitrogen, 61-7300, Rabbit polyclonal	1:100
KRT14	EMD Millipore, CBL197, Mouse monoclonal	1:100
NGFR	Promega, Anti-Human P75 pAb, Rabbit polyclonal	1:200
B-ACTIN	Cell Signaling, 4967, Rabbit polyclonal	1:1000
**Secondary Antibody**		
Alexa Fluor ®488, 594<br>Donkey anti Goat IgG	Jackson ImmunoResearch<br>(111301), (112406)	1:500
Alexa Fluor ®488, 594<br>Donkey anti Mouse IgG	Jackson ImmunoResearch<br>(112976), (110989)	1:500
Alexa Fluor ®488, 594	Jackson ImmunoResearch	1:500

Donkey anti Rabbit IgG	(113254), (113078)	
Alexa Fluor ®594 Donkey anti Rat IgG	Jackson ImmunoResearch (127426)	1:500
Anti-mouse HRP conjugated	DakoCytomation (P 0447)	1:10,000
Anti-rabbit HRP conjugated	DakoCytomation (P 0448)	1:10,000

Supplemental table 3: Primer overview

Primer	Forward primer	Reverse primer
Foxj1	5’-CAGACCCCACCTGGCAGAATTC-3’	5’-AAAGGCAGGGTGGATGTGGACT-3’
Scgb1a1	5’-GCAGCTCAGCTTCTTCGGACA-3’	5’-TCCTGGTCTCTTGTGGGAGGG -3’
Scgb3a2	5’-GTGGTTATTCTGCCACTGCCCTT-3’	5’-TCGTCCACACACTTCTTCAGTCC-3’
Trp63 (ΔNP63)[1]	5’-GGAAAACAATGCCCAGACTC-3’	5’-GATGGAGAGAGGGCATCAAA-3’
Krt5	5’-TACCAGACCAAGTATGAGGAG-3’	5’-TGGATCATTCGGTTCATCTCAG-3’
Krt8	5’-CTCATCAAGAAGGATGTGGAC-3’	5’-GAGGAAGTTGATCTCGTCGG-3’
Krt14	5’-CGGCAAGAGTGAGATTTCTG-3’	5’-AGGGATGCTTTCATGCTGAG-3’
Ngfr	5’-CGCTGACAACCTCATTCCTG-3’	5’-GCTGTTCCATCTCTTGAAAGC-3’
Gapdh	5’-CCTGCCAAGTATGATGACAT-3’	5’-GTCCTCAGTGTAGCCCAAG-3’

1. Zhao et al. Yap tunes airway epithelial size and architecture by regulating the identity, maintenance, and self-renewal of stem cells. Dev Cell 2014
